# The Distance Between the “Self” and the “Other” in Children’s Digital Books

**DOI:** 10.3389/fpsyg.2020.589281

**Published:** 2020-10-26

**Authors:** Natalia Kucirkova, Karen Littleton

**Affiliations:** ^1^Norwegian Centre for Learning Environment and Behavioural Research, University of Stavanger, Stavanger, Norway; ^2^Faculty of Wellbeing, Education and Language Studies, The Open University, Milton Keynes, United Kingdom

**Keywords:** reading, theory, IDE, socio-cultural, technology

## Abstract

This conceptual paper contributes toward our understanding of the underlying mechanisms in children’s understanding of self and the other with media. We synthesize diverse bodies of literature, concerned with children’s reading with digital and traditional (print) books, to explicate the parameters that may, in part, explain positive learning outcomes and further illuminate the patterns across various measures. We propose the “Distance Model,” which suggests that a child’s interest in a reading activity depends on its proximity to the child’s funds of identity (Esteban-Guitart and Moll, 2014). The closer the proximity, the more salient the impact on the child’s cognitive understanding and sense of belonging. The familiarity of the reading content and the relevance of the reading medium for a child’s personal life can be evoked through a number of reading strategies and design techniques, which we discuss in relation to children’s literature and the contemporary design of children’s interactive e-books. We conclude with some suggestions regarding future applications of the Distance Model in children’s media research.

## Introduction

Since the accelerated adoption of media worldwide, the discussion of children’s reading on screen has carried considerable distortions of the correlation-causality relationship in both popular and scientific discourse. Children’s use of technology has been blamed for causing a number of broader societal issues, including, for example, loneliness (Turkle, 2017) or unhappiness (Twenge, 2017) and there has been a lack of nuanced discussion about the diverse uses of media in families (for a critique see e.g., Livingstone and Helsper, 2008; Przybylski and Weinstein, 2019). Significant progress has been made in research, with interdisciplinary studies evidencing the complexities of how children’s cognitive, emotional, and social outcomes correspond to the use of diverse media in a variety of everyday contexts. Such progress has, we have argued previously (Kucirkova, 2019), been enabled through interdisciplinary, cross-cultural conversations and considerations of the relationships between individual children’s learning outcomes, individual characteristics and the design features of media. An area that needs much further development concerns the *theoretical advancement of children’s use of digital books*, and the implications that may be taken from empirical studies to the wider field of children’s interactions with technologies and social resources. In this conceptual paper we argue that the theoretical significance of diverse responses to individual features of digital books can lead to new insights in existing interpretative models of children’s learning. In particular, e-reading studies could push the boundaries of the interconnected fields of psychology and education in ways that broaden current understandings of learning. We posit that the study of *distance* between the “self” and “other” on the identity level, and the distance between “familiarity” and “unfamiliarity” on the cognitive level, could provide illuminating insights that can be applicable to future research strategy and design of children’s digital books. Although we present the two levels separately, they are mutually constitutive and exist in interplay, so they should be considered in tandem for future studies.

The argument is structured in three parts: first, we review the literature relevant to identity/cognitive distance, which we divide into three conceptual areas: psychological, phenomenological and chronological distance. Second, we provide an overview of the key theoretical concepts that substantiate the underlying mechanisms that enhance learning. Third, we draw on examples from children’s literature and e-book research to exemplify how the focus on the distance between the “self” and “other” and between “familiar” and “unfamiliar” stories, can push the boundaries of research with children’s digital books, and technology use more broadly.

Our intention is to generate discussion and new thinking, rather than attempt to summarize the comprehensive work concerning identity/cognitive research (for an overview see Garton, 2008; Schwartz et al., 2011). In accordance with the tradition of conceptual papers (see Beins and Beins, 2012), we discuss empirical studies with an emphasis on the evidence for grounding an opinion that explains new parameters and assigns a fundamental role to theoretical concepts, which are “scattered” across in the literature but that have not been related to each other and the empirical observations before. Within children’s media studies, we focus on children’s stories and digital books as a context that can contribute to the development of an understanding of the learning processes involved in children’s engagement with digital books and technology.

## A Short Note on Terminology

The terms “media” and “technologies” are often used interchangeably but they are not the same: technologies are the tools, such as smartphones or tablets, while media refers to the vehicles of engagement with technologies, using a range of communicative/literacy/textual practices, such as apps, e-books, computer games or films (see Marsh et al., 2005; Burnett et al., 2016). In this article, we selectively focus on a specific type of media: children’s books, e-books and digital books. We use the terms *digital books* to refer to digital versions of children’s books that combine images (photographs or illustrations) and text to engage children in a language-stimulating, aesthetically pleasing cultural experiences. Children’s digital books provide rich multimodal, multimedia literacy experiences through making it possible for the child to directly interact with the story characters on the digital screen by moving them across the page, hearing the book speak to the child, play songs and tunes (Ozturk and Hill, 2018). Thus, digital books are not the same as e-books, which are typically paper books translated into a digital format, without any interactivity or design adjustments (for example a novel in a PDF format read on a PC is an e-book).

The texts carried by children’s (e-)books can be fictional or non-fictional and we focus this conceptual analysis on fictional texts, which carry a story written in a narrative form. It is also important to underscore that such narratives are not neutral—they are shaped by and “carry” the psychological perspective(s) of those who developed them. Therefore, to turn a narrative into a different medium (for example, a story that first appeared in a board book into an interactive e-book), requires a translation of both the discourse (the way the story is told) as well as of the story (the sequence of events). It follows that digital stories need to go beyond the surface translation of print stories and adjust their discourse if they are to become successful in innovating the digital landscape of stories (Ryan, 2009). Lastly, the activity that stories and narratives support is that of reading and writing, or literacy. We approach literacy as plural “literacies” that are constituted through an embodied interaction with communities and everyday practices (Pahl, 2014) and that are contingent on the power dynamics and possibilities of place (Comber, 2015).

## Learning: Definition

Children’s reading experiences are typically mediated by parents, teachers, or other adults reading together with the child. But these experiences can also be mediated through prompts and design features embedded inside digital books. The dual human and material mediation, with linguistic and visual prompts, gives rise to dynamic interactions. Such interactions have been recognized, with children’s print books, as events that happen between readers and texts (Rosenblatt, 1978). When children encounter new information about self or other, they stretch their current understanding beyond what they already know, that is they *learn*. The present, (lived by the reader), and the past or future (depicted in the story) and the “here” experienced by the child and the “there” imagined or implied by the story events, need to be traversed through the act of reading. This gives rise to a learning opportunity, and this learning opportunity can be theorized as a practice of (re-)negotiating and exchanging meanings, internally and externally and thereby approximating the lived reality of two or more human minds and bodies. Neo-Vygotskian scholars, for instance, conceive of learning and meaning-making in terms of dialogue (e.g., Mercer, 1994). We work within the neo-Vygotskian tradition, according to which a dynamic learning process is usefully characterized within socio-cultural theories that emphasize the participatory and pluralistic ways of negotiating meanings and understandings (see Wegerif, 2007). Adopting a neo-Vygotskian stance on learning, we focus on the distance a learner needs to “travel” to reach the understanding of the adult/more-advanced peer. It is this focus that, we argue, provides the ground for innovation in research concerning children’s reading with digital books and that raises the fundamental moral question of how we, as educators and educational professionals, work with difference and alterity (see Baudrillard and Guillaume, 2008).

## Theoretical Framework

Theories can help us better understand the complex interactions between the technological and human aspects of reading and it is important that we situate this conceptual piece within an explanatory theoretical framework. The theory that frames this paper, and the perspective that shapes the first author’s most recent work on children’s digital books, is that of socio-materiality. Socio-materiality “involves attending to other kinds of relations: from the physicality of digital devices (e.g., their interactivity and the “screen-ness” of screens), through to the intended and unintended affordances of apps” (Burnett and Merchant, 2019, p. 265). The techno-social (or socio-material) entanglement implies that media can be used to strengthen literacy pedagogy and literacy pedagogy can strengthen digital media; one necessitates the other for securing children’s rich learning experiences. The socio-material perspective supports the notion of child-content-context-community interrelationships practices (Kucirkova, 2019), that is the combination of children’s characteristics, context of reading, content of the text and the community reading practices, that *together* shape children’s lives. What socio-materiality does not specify, however, are the mechanisms through which the context, content and individual child’s characteristics interact for stronger or weaker relations. This is, we argue, the gap that can be addressed by a consideration of findings that are connected to the theoretical basis of “distance”.

### Distance

A physicist’s understanding of distance is that of a “separation in space of the locations of two objects” (Swyt, 1992, p. 115), which, when it becomes dynamic, can be calculated as the rate of speed multiplied by time. Our understanding of distance in children’s literacies is less mechanical. To simplify, distance in children’s stories can be either experienced on the existential, identity level, or on the cognitive level of perceived awareness. Both levels are connected and inseparable. The former, identity-related distance, focuses on the gap between the “self,” the reader, and the “other,” the story character represented through the author’s work. The cognitive distance relates to the gap between familiar and unfamiliar experiences lived by the reader and depicted by the story author. Such a conceptualization of distance in children’s stories gives rise to three types, or three levels, of possible distances: (1) the psychological distance; (2) the phenomenological distance and (3) the chronological distance. These conceptual distances are realized in diverse forms in children’s literature and they can be traced back to some of the landmark studies in children’s reading and learning.

## The Psychological Distance in Children’s Texts

**FIGURE 1:**
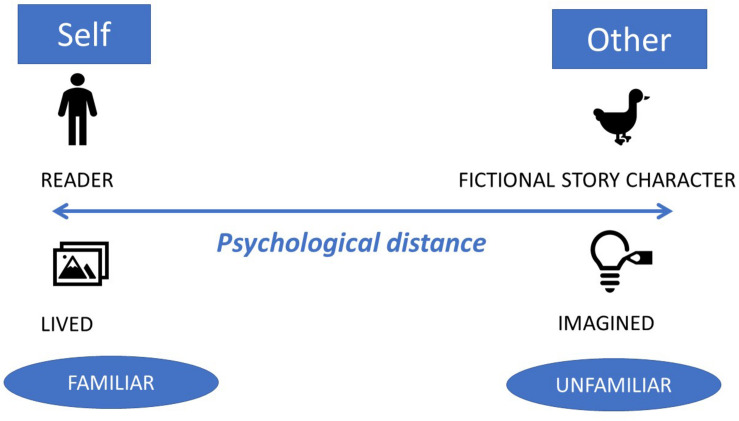
Schematic representation of the psychological distance in stories.

The psychological distance between the “self” and “other” in texts has been researched in relation to the use of linguistic cues that help readers orient themselves in text. One such linguistic tool is the use of the generic “you,” that “allows the individual to construct a generalizable lesson surrounding their experience that extends beyond the self, thus enhancing psychological distance and promoting meaning making” (Orvell et al., 2019, p. 184). Addressing readers as “you” enables them to relate the text information to their own self and build rapport with the text. The achievement of such psychological distance helps with children’s processing of the difficult emotions that they experience in an immediate situation (Orvell et al., 2017). Another way of tapping into the psychological distance in reading is to evoke self-referencing through the inclusion of the reader’s own self as subject in the text. In this instance, relatedness is established through explicitly naming the reader by name or using first personal pronouns (me, myself and I). The use of such self-referencing was tested by Turk et al. (2015) in relation to literacy attainments in experiments with 7–9-years-old and was shown to increase children’s writing and spelling skills. The self-referencing advantage extends beyond literacy benefits to increased memory effects (Cunningham et al., 2014), and has been documented in children as young as three-and-half-year-old (Ross et al., 2011). The psychological distance, then, can be bridged not only by the reader but also by the author. More specifically, the author can call for active reading through the use of “you” or through the recall of personal memories in the reader. Authors can use various techniques to support readers’ navigation of the psychological distance and readers move along the spectrum of lived and imagined stories as they navigate texts. This theoretical concept is schematically represented in Figure 1.

A fitting example that exemplifies how the two poles of the cognitive distance between the reader’s lived experience and the imagined experience of the story character become approximated is that of personalized books. Personalized books are books that have been customized, either commercially or by the readers or their relatives/friends, based on the reader’s personal information. Personalized books can be both digital or print-based; what matters from the distance perspective is whether they are personalized or not. For example, the publisher Wonderbly Ltd., uses the letters of a child’s name to customize a story about a child who lost her/his name. The story plot concerns a child looking for the letters of his/her name, which s/he finds at the end of the story. Personalized books challenge what children don’t know to a lesser extent than non-personalized books because they provide familiar clues and evolve around familiar scenarios, or at least scenarios that involve story characters and settings the child is familiar with. This lowers the threshold for participation and invites the children to see themselves in a world they are already part of. With a metaphor based on the notion of “distance,” the child readers are not traveling to meet another story character but rather they meet themselves, they stay in the destination of the familiar story, so to speak. Whilst this has its benefits, it also has significant limitations. On the positive side, personalized books can be used as tools for empowerment, which can be used for therapeutic purposes, such as raising children’s self-esteem, confidence and enjoyment of reading (Demoulin, 1999). On the other hand, the reading of children’s personalized books was found in our own prior work to be correlated with children’s self-referential speech, indicating a heightened focus on self (Kucirkova et al., 2014).

The distance between fictional and personal worlds can be enlarged or reduced through prompts that are perceived not only on the cognitive and linguistic levels, but phenomenologically, that is, through the whole body.

## The Phenomenological Distance

Phenomenology is a broad term but in the study of literacies and literature it is defined as the “study of the phenomenon,” “that which appears,” that is to say, an occurrence perceptible by the senses’ (Mildenberg, 2017, p. 13). Children’s books “tap into” children’s visual perception through images and illustrations, while audio stories stir children’s imagination through the auditory system, and multimedia stories typically engage visual, audio and tactile senses. Merleau-Ponty’s (1982) account of phenomenology is concerned with the lived body between ideas and objects and the meanings that the body holds. It positions childhood as an embodied experience (Welsh, 2013), whereby language is not restricted to linguistic or cognitive studies but expanded to a corporeal language of being. Studies show that contemporary readers move between various story formats and story representations, thus accommodating the multiple ways of being.

**FIGURE 2:**
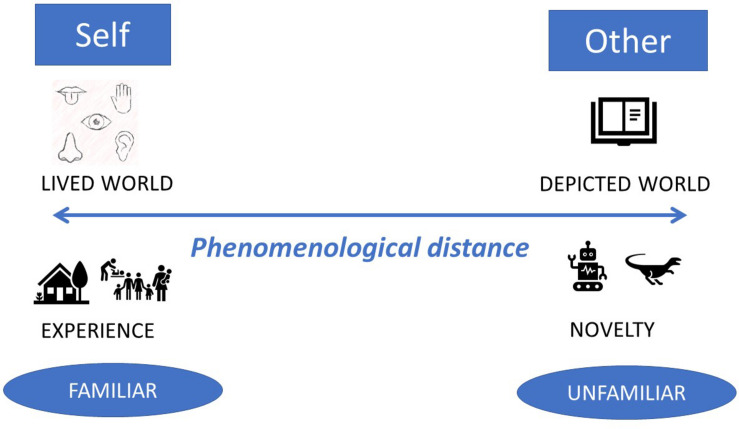
Schematic representation of the phenomenological distance in stories.

For example, Rowsell (2014) documented how students traveled between print, videos or blogs to connect their narratives in classrooms, and Hackett (2016) described the experience of 2- and 3-years-old connecting their remembered and immediate experiences of stories with the unfamiliar stories represented as objects in museums. The distance in the phenomenological sense thus refers not only to the gap between the physical experiences of the reader and the physical object of a print or digital book, but also to the depiction of these experiences in various story genres and through various story techniques. In terms of story content, the distance is larger when the story illustrations evoke environments further from those that the child has seen or experienced before (as in, for example, fantasy literature) and the distance is shorter when the story representations approximate the lived experience through story plots and story scenes familiar to the reader. In terms of story formats, the embodiment possibilities with digital books, and their links to lived experiences, are even more diverse than with analog books as visual perception is extended to audio perception and tactile feedback from the book (for example touching the story character makes it change color or perform an action). Although empirically yet to be verified, some researchers hypothesize that this physically close relationship between the story characters and the child’s body (finger moving on the screen) impacts children’s understanding of the characters’ emotions and children’s empathy (Zhao and Unsworth, 2016). The phenomenological distance in children’s stories is schematically represented in Figure 2.

## Chronological Distance

In Bakhtin’s (1981) chronotope theory, the space in the story corresponds to the movement of time, while the time *places* the story characters’ actions and movements. In his study of literary narratives, Bakhtin (1981) suggested the use of the term “chronotope,” derived from the Greek “chronos” that stands for time and “topos” that stands for place, to unify time and space in a deep entanglement of time-space as one word and phenomenon. Given that in chronotopes time and space are interlinked, the Bakhtinian account provides a powerful explanatory structure for the distancing necessary to bridge the time depicted in books and the time experienced individually by the readers. As Johnston (2002, p. 137) puts it, the concept of chronotope: “helps us to read beyond the mechanics of ‘setting’ and to rethink depictions of narrative time-spaces in terms of being essentially ideological, that is, as subjective, changeable, multiple and dependent on the position of the observer.” In the case of children’s literature, the observer is the child, who interprets the words and images in picture books through her own experience. As literary scholars argue, the distance between the chronotopes of literature needs to be supplemented with a contemporary context that connects the experiences and actions of the story characters *with* the temporal ordering in stories (Scholz, 1998). The young reader connects to the narrative structure of stories on the temporal level in terms of their own personal story and the experience of time in the child’s immediate environment. For the latter, there are known variations between how children from different cultures respond to the classic temporal arrangements of story beginning, middle, and end (Peterson and McCabe, 1994). This is because despite shared clocks and biological rhythms, the experience of time is very individual (Leaton Gray, 2017) and depends on a range of socio-material factors (e.g., the waiting time in a doctor’s office might be the same length for everyone but is perceived differently by each person). From the perspective of chronological distance, the distance between the story and the reader can be fairly abstract in terms of “lived time,” which is the time experienced socially, and the time lived individually by the story characters in the text. It follows that for a meaningful reading experience, authors need to draw links between the collectively shared and the personally experienced time-space. In historical or futuristic novels, this is achieved through various visual and linguistic techniques.

**FIGURE 3:**
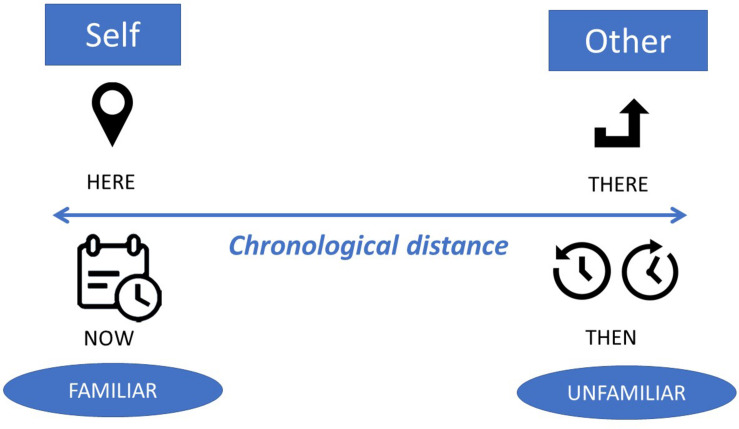
Schematic representation of chronological distance.

For example in wordless picture-books, “without integrating a clock, a calendar, or a similar device, precise time and passage of time is difficult to represent in a wordless picture book” (Beckett, 2013, p. 128). Given that children have a very different understanding of time than adults and given that most children’s books are written by adults for children, the bridging across the time-space often happens through literary techniques that reverse adult-child roles (O’Sullivan, 2005). Readers cross, to a greater or lesser extent, the chronological distance as they negotiate their understanding of the stories’ time and space. Figure 3 provides an illustration of this journey.

Our summary of the research that could be attributed to the psychological, phenomenological and chronological distance in children’s literacies is necessarily short in this paper but discussed more fully in Kucirkova (2021). The summary here, nevertheless, serves as a reference point for understanding the Distance Model.

### The Distance Model

**FIGURE 4:**
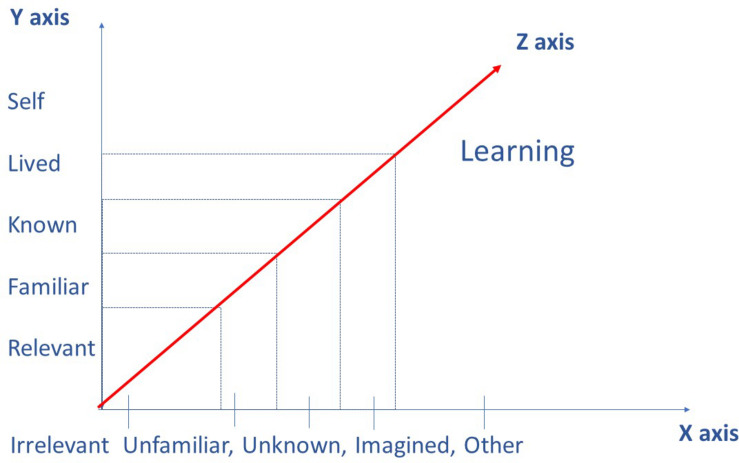
A schematic representation of the Distance Model.

For future replicability and expansion of empirical findings, there needs to be a model that viably explains the mechanisms of observed phenomena and that provides a common language for conceptualizing future studies in children’s interactions with digital books and children’s technology more broadly. The Distance Model is not an analytic framework, but a conceptual tool that can be used to explain existing findings and propose new directions for future research. The Distance Model positions learning as a spectrum, or as being like an elastic band that is not fixed but that can be increased or decreased, depending on how close the learner is psychologically, phenomenologically or chronologically positioned to the other. The foundation of the model is that of an optimal negotiation of psychological, phenomenological and chronological distances between the familiar and the unfamiliar experience/event. In this depiction (see Figure 4), the self, and the familiar world the self stands for, is shown along the *Y*-axis and the other, and the unfamiliar, new, world the other stands for, along the *X*-axis. The *Z*-axis (depicted in red) in the figure represents the optimal meeting point between these two poles, which is the point, where learning happens.

Why does the Distance Model position learning as a measure of the distance between the familiar self and the unfamiliar other? There are several plausible explanations for why the self-other negotiation supports learning, and these explanations have been gauged in terms of the “relevance” or “learners’ identification” with learning materials. These terms, however, only relate to surface characteristics that do not account for why learning occurs. To understand the “why” of self-other distancing, we need to turn to theory. For purposes of this discussion, we selectively focus on the funds of identity theory (Esteban-Guitart and Moll, 2014) as it affords a powerful characterization of the identity formation between self and other, that can provide the foundations for the Distance Model.

## Why Does Learning Occur When Bridging the Self-Other Distance?

All readers and writers have their own “funds of knowledge,” that is a collection of skills, wisdoms and understandings that they use to define themselves and the world around them (Moll et al., 1992). A dialogue is the dynamic process of opening up to the voice of the other, which widens an individual’s perspective and prompts an individual to not only internalize but also externalize his or her understanding. Wegerif (2007) conceives of dialogue as occurring in a dialogic space, which further extends the meaning-exchange on a cognitive level to a whole-body resonance of feelings and thoughts. A dialogic space connects to Merleau-Ponty’s phenomenology as well as the socio-material notion of entanglement between humans and non-humans. A dialogue in a dialogic space is not only about exchanging individual and collective knowledge in a narrow, educational, sense but also the ways of being and doing in a socio-historical sense (Wegerif, 2011). The cultural understanding of meanings and sense-making that all children have and all children bring to their learning environments is known as “funds of knowledge,” originally Moll et al. (1992) and recently expanded to funds of identity by Esteban-Guitart and Moll (2014). Predicated on Vygotskian thinking, Esteban-Guitart and Moll (2014, p. 37) define funds of identity as: “historically accumulated, culturally developed, and socially distributed resources that are essential for people’s self-definition, self-expression, and self-understanding. In other words, the term ‘funds of identity,’ which we are using here, denotes a set of resources or box of tools and signs.” Building on this, we see the distance between self/familiar and other/unfamiliar played out in terms of the difference between the historically accumulated resources implicated in the negotiation/(joint) construction of selfhood and the reading approach in question.

This points to a balancing process that rests on shortening and enlarging the distance between the two poles, as represented by the *Z*-axis in Figure 4. Theories of the “dialogic” and “dialogic space” have been embraced by many educational researchers, who have developed a body of empirical research that documents the various conditions under which learning occurs (see e.g., Ludvigsen et al., 2019). An interesting point of convergence in the studies, from the Distance Model perspective, is that of the conditions that contribute to optimal learning where meanings between self and other are exchanged, challenged and acquired. In some circumstances, the distance becomes too large and in others it can be easily bridged with some support. This brings us to the idea of thresholds and the educational theories that specify the learning conditions in which children appropriate new knowledge.

## How Can Distances Be Bridged?

Moving on from the account of how dialogue and dialogic space rest on the notion of distance between the “self” and the “other,” we now use the Distance Model to explain how the psychological, phenomenological and chronological distances can be bridged in the context of children’s digital books. With a nature- or biology-oriented perspective that emphasizes the role of genetic factors, and is different from the socio-cultural view on learning, the threshold when the “self” (or *a priori* cognitive schemas) become established is an important milestone for acquiring new knowledge (see e.g., Piaget, 1950; Bogard et al., 2013). As a good example, take the study by Sui and Zhu (2005), in which they tested 4, 5, and 10-years-old children’s response to cartoon figures with the children’s own or another child’s face. The 5 and 10-years-old remembered more objects associated with the figure showing their own face, but for the 4-years-old, there was no such “self-advantage.” While from the nature-oriented developmental perspective, there is a threshold for when self-referential encoding turns into a learning advantage, from the educational perspective, the learning tipping points happen at less predictable stages. Adopting a nurture-oriented perspective that emphasizes the role of environmental factors, researchers Adams et al. (2015) introduce the terms of “liminal spaces” and “threshold concepts.” Drawing on the time-space boundaries in learning elaborated by Maaninen-Olsson and Müllern (2009), Adams et al. (2015) use threshold concepts to indicate that the progress to an individual’s understanding involves crossing a boundary that is not inherent to the individual but to the learning environment. A threshold concept is conceptualized as a doorstep, which, when crossed, allows students to reach a higher level of understanding but if not understood, leaves students disengaged and unable to progress their learning (Cruz et al., 2016). It follows that classrooms are conceptualized around shared values and acquisition of new knowledge and facts, and this focus is actively fostered through social upbringing and collective perceptions.

From a socio-cultural perspective, “dialogic space” in contemporary classrooms is, as envisioned by Wegerif (2013), a fertile ground for learning and problem-solving when there is a difference of ideas, perspectives and understandings. This opens up a space of possibility, full of potential for exploration and dialogue. It is thus not similarly/proximity of viewpoint(s) but rather differences in perspective, which leverages the building of understanding. Whether we place a more nature- or nurture-oriented lens on these approaches, we see that both converge on the question of boundaries and that these boundaries relate to how big the gap between familiar and unfamiliar is. In this respect, the Distance Model provides a thinking tool for depicting the learning process as a sliding scale, which increases or decreases depending on the learner’s dispositions and the environmental scaffolds available to this learner. If we map these insights onto the skeleton of the Distance Model, we find a neat mapping on what Vygotsky (1978a, original in 1935) described as the Zone of Proximal Development: “the distance between the actual developmental level and as determined by independent problem solving and the level of [potential development as determined by problem solving under adult guidance or in collaboration with more capable peers p. 86.” Learning is thus the distance a learner needs to travel to reach the understanding of the adult/more advanced peer (this peer could be the adult or the digital book designed to support the individual).

Vygotsky’s ZPD is helpful, and it specifies the departure and arrival areas, metaphorically speaking, but it does not focus on the joint journey that needs to be undertaken by both the self and the other. Vygotsky wrote that “Every function in the child’s cultural development appears twice: first, on the social level, and later on the individual level; first, between people (interpsychological), and then inside the child (intrapsychological) p. 57.” This applies equally to voluntary attention, to logical memory, and to the formulation of concepts. All the higher functions originate as actual relations between human individuals (Vygotsky, 1978b, p. 57). In Vygotsky’s writings, learning is positioned as a cognitive trip from the collective to the individual. Yet, neo-Vygotskian scholars argue that intramental and intermental learning need to be combined, in the activity of “interthinking,” which often leads to better results than individual thinking (Littleton and Mercer, 2013). Adopting this perspective, we argue that the focus on the distance, on the journey, so to speak, allows us to forge more accurate results in terms of innovative research focus.

In the following section, we discuss these insights in relation to the type of distance (psychological, phenomenological and chronological), and we illustrate how the self/familiar-other/unfamiliar distance is addressed in practice, design and research of digital books. We conclude each example with recommendations for future approaches to push the boundaries of current knowledge in children’s digital books.

## Bridging the Distances and Advancing Future Research With the Distance Model

Prior research has progressed our understanding of expected outcomes when learners interact with digital books and make progress in vocabulary learning (e.g., Korat and Shamir, 2012) or story comprehension (Dore et al., 2018), as well as of the obstacles that learners face in assimilating meaning from highly interactive digital books that distract from the main story content (Takacs et al., 2015). The empirical advancement has also identified the specific features of digital books that hold the promise of digital books to innovate children’s reading (Bus et al., 2020), or the specific mediation strategies used by adults to facilitate children’s comprehension (Chen et al., 2020). Studies also show that considerable individual variation exists in respect to these findings, which is attributable to cognitive capabilities, including children’s executive functioning (Richter and Courage, 2017) or attention (O’Toole and Kannass, 2018). What is less known is the joint journey that “self” and “other” can undertake to expand the boundaries of their familiar and unfamiliar story worlds.

## Psychological Distance: Future Research on Children’s Personalized Books

A future focus that centers on the *distance* could expand the research on personalized books to more imaginative story-worlds and more diverse types of personalized books. This would be particularly useful for exploring the reality of difference and empathic responses to others who are markedly different from self. How close the distance between lived and imagined reality should be in children’s books is disputed but there is no doubt that “these images form and inform the unconscious substrate, or what cognitive theorists call the schemas, that we use to process everyday experience (…) creating a sense of obviousness that may have extended consequences for children’s developing understanding of gender and race in certain cultural contexts” (Coats, 2019, p. 364). Children’s authors and illustrators typically capitalize on children’s imagination by depicting fantasy story-worlds with supernatural creatures that allow self-projection and that stimulate children’s creative thinking (Nikolajeva, 2015). Future research on personalized books could thus probe various kinds of fantasy worlds, such as children’s avatars, immersion in alternative worlds with supernatural creatures or children from other cultures and story worlds the child is unlikely to experience because of their living situation. Such titles would still comply with the aim of empowering young readers, with for instance lending young reader superpowers or enabling them to see a natural world they cannot visit because of an illness, displacement or financial resources. Digital books could expand this area, for example if they employ virtual and augmented reality to immerse children in alternative story worlds, such as the recently launched “The Case of the Missing Cleopatra” by AR Market. Empirical research is significantly behind the technological advances in personalized books and the distance perspective could increase researchers’ interest in the “self” and “other” relationships in children’s contemporary reading. It is necessary to establish the techniques that authors/publishers/designers could use to increase or decrease the self-other distance and for which outcomes. Literature with non-personalized analog books shows variation in the optimal distance between the story character and the child’s persona: blurring fantasy with reality in anthropomorphic books was found to be detrimental to children’s knowledge about the animals (Ganea et al., 2014) in one study, but did not show any difference to children’s factual understanding in another study (Geerdts et al., 2016).

There is a very close link and a functional overlap between autobiographical memory and theory of mind (Spreng et al., 2009) and personalized books are likely to tap more directly into the autobiographical memory domains than non-personalized books, especially if they are produced digitally with photographs or videos showing children’s lived experiences. This could be employed for teaching children self-regulation and empathy for others but also for learning new concepts. For example, one could imagine radically impersonal texts such as non-fiction books about animals, with personal cues such as a child’s name or photo embedded in the book. The “self” would be stretched from the familiar position to that of others, as suggested by socio-cultural, neo-Vygotskian, scholars. The “self” would bridge the conceptual but also the material, familiar space and thus advance socio-material forms of being in the world.

## Phenomenological Distance: Future Design of Children’s Digital Books

Phenomenological distance relates to the notion that the proximity of a story character to a reader needs to be tempered not only visually (through texts and illustrations), but through the engagement of all senses. A broad look at the children’s literature suggests little innovation in the area, so far, with the vast majority of books offering content through visual and tactile engagement (in the form of print or digital books). However, bridging the phenomenological distance would mean engaging also the auditory, olfactory and gustatory sense, and it is with multisensory books that the design of children’s digital books could innovate the field.

The representation of stories can be in various formats and each format can afford a distinct experience with so far, little understood benefits. For instance, audiobooks leave the visual depiction of stories entirely to listeners’ imagination and they extend the reading experiences in classrooms (Larson, 2015). As a new digital literary experience, they create new learning experiences that, arguably, constitute a new form of reading: “reading by listening is a specific form of semantic listening separate from other forms of semantic listening such as those involved in conversations or consumption of vocal music” (Tattersall Wallin and Nolin, 2020, p. 471, 472). While there are known gender and age differences in the reading of traditional paper-based books, Tattersall Wallin and Nolin (2020) found no significant gender or age differences in the reading of audiobooks. More research is needed to explore the ways in which non-visual engagement with stories might address long-standing discrepancies in learning and expand the possibilities of what reading can achieve.

The Distance Model positions learning as the meeting place of the familiar/unfamiliar self/other, which implies a travel on a two-way roadway. The innovation necessary to bridge the distance should thus not be focused solely on replicating a lived experience with technologies, but also on enabling the reading technology to shape the lived experience. This opens up possibilities for innovations that could initiate a substantial structural change to how we understand the role of stories and what reading might look like. For instance, the use of edible materials has not been traditionally considered as part of children’s reading experience, but Alaca (2019) comprehensively summarized the benefits of edible reading for expanding children’s enjoyment of texts and tapping into learning domains that combine awareness of healthy eating with texts. Outlining the example of edible books, Alaca (2019) presents a prototype of snacks with printed letters and suggests that adding vitamin letters on edible snacks or print papers (made of rice paper) could engage children and adults in an enjoyable and health-promoting reading experience.

Another book format that carries a significant potential to expand the repertoire of children’s reading experiences, are olfactory books. Olfactory books engage children’s sense of smell through the release of scents or smells in relation to the concepts depicted on individual pages. Such books are, at the time of writing, in the prototype stage by the first author, so their learning potential can only be hypothesized, but the idea indicates the range of possibilities that are untapped in the current predominantly visual focus of children’s literature. Crucially, if we are to bridge and challenge the familiar space of current socio-historical and socio-cultural understanding, then this bridging does need to involve the whole, multisensory nature of a human experience.

## Chronological Distance: Future Studies in Children’s Literature

The distance between the “here” and “now” and the “there and then” is bridged successfully when readers immerse in a story, and when they identify with the story characters and engage with the content when the reading experience has ended. Such a reading experience is that of reading for pleasure (rather than for finding specific information or for a specific purpose and it is characterized by desire, diversity and delight (Cremin, 2008). The space-time boundaries shift as the reader navigates the story landscape and provide opportunities for reminiscing and revisiting the readers’ own memories of a lived experience, as well as the reader’s imagination and projections for the future.

The temporal and the spatial coordinators in books have an “inner diversity” (Luo, 2017), and the extent to which this diversity is taken up by readers depends on readers’ agency. A central issue with the Distance Model becomes that of *agency*, where the reader decides himself or herself how a story continues. Such volition is supported through readers’ own dispositions, such as their reading ability or interest and social capital, such as access to books (Love and Hamston, 2003). Authors, illustrators and designers introduce their own agency into a story through specific story-telling techniques, which have been studied in detail by literary scholars. Luo (2017), for example, shows how the popular story of Narnia does little to reduce the distance between Aslan and the reader, thus reducing the reader’s agency: “meanings and values are complete and determinate, as is specifically presented in the stories as the absolute faith and unconditional obedience Aslan demands. The epic chronotope does not open to human initiative or personal judgment. It demands to be evaluated in the same way by all. This immanent characteristic of the epic chronotope determines that only the role-defined characters—who are given limited inner depth and who are largely subordinate to the plot—can be compatible with it” (p. 76). In contrast, in analysis of the chronotope in three popular children’s Scandinavian picturebooks by the author Dahle and illustrator Nyhus, Krogstad (2016) highlights the close proximity between the time-space experienced by the depicted story characters and the intended reader. This gives rise to the child’s agency because the child is portrayed “as an independent individual, with the potential to act, develop, and change, which may also actualize ethical aspects connected to the child as an actor, such as the way in which the child relates to others and communicates with other generations” (Krogstad, 2016, p. 10). A methodological focus on readers’ and authors’ agency is essential for nurturing these relationships. There is, however, no consensus on how to study children’s agency in relation to reading.

As outlined in Kucirkova (2018), agency can be studied in relation to three indicators: behavioral indicators of children’s control during the book reading (for example how the child holds the book, where the child looks, how the child turns pages), adults’ perceptions of reader identities afforded by the content and format of books (this is the so-called social agency), and through specific multi-media and interactive features that are embedded in the books and allow readers to make choices. The latter can, for example, be manifested through books where children can choose the story characters or story endings, and in this way mediate themselves the distance between the “here and now” of their lived experience and the “there and then” of the story. More research into how the concept of distance can be applied methodologically, and how to study children’s agency with print and digital books, is needed.

In conclusion, the children’s digital books research can be advanced with the Distance Model. This has yet to permeate widely the field of children’s media. The distance between the familiar, me-oriented, world of stories and the novel, depicted, world of stories, is the space between reality and the interpretations of reality. By extension, structuring future studies in children’s media around the idea of distance is part of researchers’ agency to trouble the current interpretations of what is possible when the distance between the “self” and the “other” becomes their joint horizon.

## Author Contributions

NK developed the argument, framework, and figures. KL added perspectives drawn from the literatures concerning funds of identity and dialogic space. Both authors contributed to the article and approved the submitted version.

## Conflict of Interest

The authors declare that the research was conducted in the absence of any commercial or financial relationships that could be construed as a potential conflict of interest.
